# Polycyclic Aromatic Hydrocarbon (PAH) Contents of Four Species of Smoked Fish from Different Sites in Senegal

**DOI:** 10.1155/2023/2931615

**Published:** 2023-05-13

**Authors:** El Hadji Moussa Diop, Bou Ndiaye, Alioune Sow, Falilou Mbacké Sambe, Mamadou Sall, Mamadou Saliou Thiam

**Affiliations:** ^1^Water, Energy, Environment and Industrial Processes Laboratory, Polytechnic School, Cheikh Anta Diop University, Dakar, Senegal; ^2^School of Industrial and Biological Engineering, Dakar, Senegal; ^3^Center for Studies on Food Safety and Functional Molecules (CESAM-RESCIF), ESP-UCAD, Dakar, Senegal; ^4^Department of Agronomic Sciences, Aquaculture and Food Technologies, Gaston Berger University, Saint-Louis, Senegal

## Abstract

Polycyclic aromatic hydrocarbons (PAHs) are compounds resulting from any incomplete combustion process. These are pollutants that have proven toxicity due to their carcinogenic nature and can contaminate food during traditional smoking methods. Their highly toxic effect on human health requires monitoring of their levels in food products and the development of appropriate analytical methods for their determination. Thus, this study was conducted to assess the level of PAHs contamination of four (4) species of smoked fish (*Arius heudelotii*, *Sardinella aurita*, *Ethmalosa fimbriata*, and *Sardinella maderensis*) which were sampled in seventeen (17) localities in Senegal. The compounds targeted in this study were benzo(a)pyrene (B(a)P), benzo(a)anthracene (B(a)A), benzo(b)fluoranthene (B(b)F), and chrysene (Chr). The QuEChERS method was used for the extraction of PAHs, and their contents were quantified by gas chromatography (GC) coupled with mass spectroscopy (MS). The validation method was performed in accordance with the French standard NF V03-110 (2010). Satisfactory linearity (*R*^2^ > 0.999), LOD (0.05–0.09 *μ*g/kg), LOQ (0.19–0.24 *μ*g/kg), and precision (1.33–3.13%) of the four PAHs were obtained. The results of analysis in the 17 localities showed that all samples are contaminated by the four (4) PAHs with great variability of the contents between the different species and their origin. The B(a)P and ∑4PAHS contents in the samples ranged from 1.7 to 33 *µ*g/kg and from 4.8 to 1082.3 *µ*g/kg, respectively. Twelve (12) samples showed high levels of B(a)P, ranging from 2.2 to 33 *µ*g/kg, thus exceeding the maximum authorized level (2 *µ*g/kg). Fourteen (14) samples showed an overall ∑4PAHS content varying from 14.8 to 1082.3 *µ*g/kg, which is above the maximum authorized limit (12 *µ*g/kg). The principal component analysis showed that *sardinella* (*Sardinella aurita* and *Sardinella maderensis*) have very low levels of B(a)P, B(b)F, B(a)A, and Chr contents. However, high ∑4PAHS contents characterize smoked fish of the Kong species (*Arius heudelotii*), from Cap Skiring, Diogne, Boudody, and Diaobé, and of the Cobo species (*Ethmalosa fimbriata*) from Djiffer. Thus, based on the authorized limits for PAHs in smoked fish, it appears that smoked fish of the *sardinella* species are less carcinogenic for human consumption.

## 1. Introduction

Fishing is one of the most dynamic sectors in Senegal as it has experienced strong growth over the past three decades. Catches have increased considerably eight (8) times in thirty-two (32) years [1]. A source of jobs, income, and foreign currency for stakeholders and governments, fishing is a real center for economic and social development. In Senegal, the average of sea fishing landings over the period 2009–2018 was estimated at 421,000 tons, 89% of which was carried out by artisanal fishing. In 2018, the total national fish production reached more than 461,000 tons [2]. Most of the catches come from small-scale maritime fishing (94.7%) [3]. It should also be noted that fish is one of the main sources of animal protein for populations, thus contributing to more than 75% of protein intake [4–6]. However, the fish is known for its highly perishable nature [7]. Indeed, it can deteriorate very quickly and become unfit for consumption and even dangerous for health due to microbial proliferation, chemical modifications, and degradation by endogenous enzymes [4]. Thus, due to the large quantities produced, the perishable nature of the fish, and the lack of appropriate conservation equipment, large quantities of fish are processed using traditional methods.

Among these artisanal processing methods, smoking occupies a prominent place in developing countries. This technique is the main and sometimes the only means of supplying populations with fish, especially when they are far from fishing sites. Smoke not only gives fish a special taste and aroma but also improves preservation due to its dehydrating and bactericidal properties [8]. However, smoke, in particular that of wood, is a vector of compounds called polycyclic aromatic hydrocarbons (PAHs), known for several decades for their carcinogenic power in humans [9]. This artisanal processing technique is generally known to generate and increase the level of PAHs in food. Moreover, many studies have shown the presence of PAHs in smoked fish [10, 11]. Today, the presence of these PAHs in food products, especially smoked fish, is a subject of major concern.

It is in this context that this present study occurs, which had as its objective the determination of polycyclic aromatic hydrocarbons (PAHs) in four (4) species of smoked fish from different areas in Senegal. Thus, the contents of benzo(a)pyrene (B(a)P), benzo(a)anthracene (B(a)A), benzo(b)fluoranthene (B(b)F), and chrysene (Chr), enacted by European Commission (EC) Regulation No. 835/2011, were estimated. A principal component analysis (PCA) was done to assess the effect of fish species, source, and technology of processing on the quality of smoked fish.

## 2. Materials and Methods

### 2.1. Biological Material

The biological material consists of four (4) species of smoked fish that were chosen from the processors. These were Kong (*Arius heudelotii*), Round *sardinella* (*Sardinella aurita*), Cobo (*Ethmalosa fimbriata*), and Tass or Flat *sardinella* (*Sardinella maderensis*). The choice of these four species was justified by the fact that they are more consumed in Senegal.

### 2.2. Sampling and Preservation

**Figure 1:**
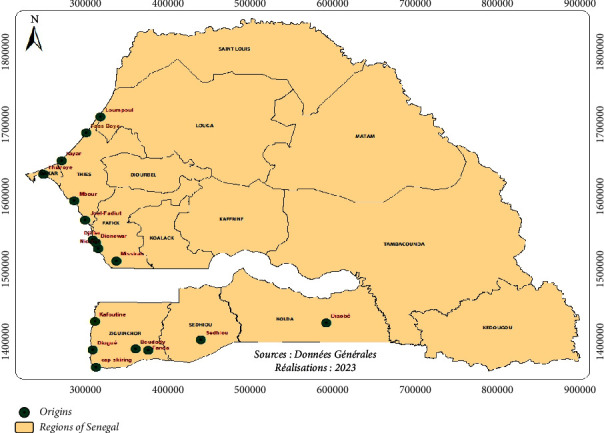
Geographic plan of the seventeen sampling areas.

Smoked fish samples were collected between February and June 2021 in seventeen (17) fish-processing sites in Senegal.  Figure 1 presents the geographical plan of the 17 smoked fish sampling sites. These sites are located on the Senegalese coast near the landing centers for artisanal fishing. Thus, for each sample, the fish were packed in bags and then stored at −4°C. Once in the laboratory, they were crushed, and then introduced into QuECherS (quick, easy, cheap, rugged, and safe) tubes and stored at −20°C in the freezer until the start of the analyses.

### 2.3. Chemicals

Chemicals and solvents used are of analytical reagent grade. The acetonitrile (99.9%) and the acetone (99.8%) were, respectively, obtained from Sigma-Aldrich and VWR Chemicals. QuEChERS extraction tubes were purchased from Thermo Fisher Scientific Inc. (USA) and consisted of 50 mL centrifuge tubes containing 4 g anhydrous magnesium sulfate and 1 g sodium acetate. The 4 standards PAHs (B(a)P (CAS No. 50-32-8), B(a)P (CAS No. 56-55-3), B(b)F (CAS No. 205-99-2), and Chr (CAS No. 218-01-9), manufactured by Supelco, were purchased as pure compounds.

### 2.4. Methods for the Extraction and Analysis of PAHs in Smoked Fish

The QuEChERS method was used for the extraction of PAHs [12]. The proposed method is fast and effective and can be successfully applied for PAHs determination in difficult matrices such as heat-treated food of animals [13]. For this, a test portion of 5 g of fish flesh was ground, homogenized, and introduced into a 50 mL QuECherS tube. Then, 20 mL of an acetonitrile-acetone mixture (60 : 40 v/v) is added thereto. The whole was stirred vigorously at 2000 rpm using a DLAB MX-S vortex (DLAB Scientific Inc.) for 30 seconds and then with an ultrasound bath for 5 minutes. After this step, centrifugation for 7 minutes at 3000 revolutions per minute was carried out, using a Medibas + centrifuge mod. 2741 (Auxilab). The supernatant was transferred to a 250 mL flask and then evaporated to dryness at 35°C using a rotary evaporator. Then, the concentrated residue was extracted with 10 mL of the acetonitrile-acetone mixture (60 : 40 v/v), and then the whole was transferred into another 50 mL QuEChERS tube and centrifuged at 2000 revolutions per minute for 1 minute. The supernatant was recovered and placed in a 100 mL flask and then evaporated at 35°C. The concentrated residue contained in the 100 mL flask was again recovered with 2 mL of ACN using a 1000 *μ*L automatic pipette. Everything was transferred using a Pasteur pipette into a vial and then frozen for 24 hours to freeze the fat. Finally, the supernatant was collected using a Pasteur pipette into another vial. The latter was placed in a gas chromatograph (GC) of the Agilent 7890A type coupled to a mass spectrometer (MS) (Agilent 5975C). The apparatus was equipped with an automatic sampler. Separation of PAHs was conducted using a 5% phenyl-methylsilicone (HB-5MS) bonded-phase fused-silica capillary column (Hewlett-Packard, 30 m *∗* 0.25 mm I.D., and film thickness 0.25 *µ*m). Helium (99.999% purity) was used as the carrier gas with a flow rate of 1.2 mL/min. The injection port was adjusted in splitless mode, and the injection volume was 1.5 *µ*L. The GC was programed as follows: initial temperature 70°C for 5 min and ramped at 25°C/min to 310°C for 13 min and allowed to stay for 10 min giving a total of run time of 28 min. The mass spectrometer (MS) was operated in the electron ionization mode, with an electron energy of 70 eV. The MS transfer line and ion source temperatures were adjusted at 310°C and 290°C, respectively.

Of the eight (8) PAH molecules recognized as carcinogenic when present in food, four (4) have been determined as benzo(a)pyrene (B(a)P), benzo(b)fluoranthene (B(b)F), benzo(a)anthracene (B(a)A), and chrysene (Chr).

In order to increase sensitivity, all the GC-MS measurements of the different samples were carried out in triplicate and the results recorded in the various tables represented the average.

### 2.5. Validation Method

The quantification method of PAHs in smoked fish samples was developed and validated according to the indications of the European Commission (no. 836/2011) and the French standard NF-V03-110 (2010). This procedure included successive steps and the determination of different parameters such as the linearity of the calibration interval, the detection and quantification limits, coefficients of variation for the repeatability, and intermediate precision tests.

The identification of PAHs was verified by comparing the retention times of each standard solution between each test. For the linearity tests, the standard stock solutions were dissolved in acetonitrile and five working solutions (0.005, 0.1, 0.2, 0.5, and 1 ppm) were prepared to distinctly construct the calibration curves. These curves made it possible to establish an adequate correlation between the areas of the peaks and the concentrations of PAHs found in the samples tested. The limits of detection (LOD) and quantification (LOQ) were calculated from the standard PAH solution by separate 3 × 10 analysis according to the repository (NF-V03-110 2010). The values of these limits expressed in (*µ*g/kg) are given by the following formulas:

(1) $$\begin{array}{c} LOD=mb+3\sigma \text{,}\\ LOQ=mb+10\sigma \text{,} \end{array}$$

where **mb** is the average of each marker PAHs content in the standard solution and ***σ*** is the value of standard deviation for the norm for each PAH marker.

Besides, the analytical stability was assigned by the analysis of a standard solution of 1 ppm by calculating the relative standard deviation of the measurements. By repeating the analysis three times per day (intraday) for three consecutive days (interday), the precision (%) expressed as a coefficient of variation (CV) was obtained.

(2) $$\begin{array}{c} CV\;\left(\%\right)=\left(\frac{Standard\;deviation}{Mean}\right)*100\text{.} \end{array}$$

### 2.6. Statistical Analyses

All analyses were injected in triplicate to obtain a good precision. GC-MS data were acquired and analysed by Agilent Chemstation software for GC (G2070BA) and a Microsoft Excel version 2016 spreadsheet. A principal component analysis (PCA), which is the most widespread of the factorial methods [14], and a numerical classification were carried out on the results of analyses of the seventeen (17) zones in order to bring out the information carried by the four (4) PAHs contained in the samples. Thus, all analyses were performed with the software *R* (version 4.1.1, 2021).

## 3. Results

### 3.1. Validation of the PAHs Determination Method

The method validation results as the limit of detection (LOD), limit of quantification (LOQ), linearity, and retention times are shown in Table 1. B(b)F, Chr, B(a)A, and B(a)P were identified, respectively, at times 22.43, 23.66, 21.22, and 27.52 minutes. The determination of the correlation coefficients ranged from 0.9994 to 0.9999. The respective values of the detection limits were 0.05, 0.09, 0.07, and 0.08 *μ*g/kg and those of quantification were 0.19, 0.24, 0.2, and 0.23 *μ*g/kg for the B(b)F, B(a)A, Chr, and B(a)P. The LOD and LOQ values were within the acceptable limits of ≤0.3 *µ*g/kg and ≤0.9 *µ*g/kg, respectively, for each of the four PAHs (B(a)A, B(a)P, B(b)F, and Chr). The calculated coefficients of variation of the repeatability tests ranged from 1.33–2.25% to 1.95–3.13%, respectively, for intraday and interday. Corresponding results for the PAH4 are presented in Table 2. The tests of reproducibility corroborate the results' repeatability. Indeed, the coefficients of variation obtained were less than 15%. The results of these validation tests are in adequacy with the European Commission (EC) standard no. 836/2011. The data obtained from the validation of the method in this study confirm that GC-MS is satisfactory for the analysis and detection of PAH4 in smoked fish samples.

### 3.2. PAH Content in Smoked Fish Samples

The results from the analyses are presented in Table 3.

The results showed a great variability of the contents between the various species and according to their origin. The B(a)P molecule, representing 40% of the total carcinogenic risk attributed to PAHs [16], is present in all the samples analysed with sometimes levels that exceed the authorized limit (2 *µ*g/kg). Thus, it appears from these analyses that the samples taken in the areas of Dionewar (2.3 *µ*g/kg), Fanda (2.2 *µ*g/kg), Joel (3.9 *µ*g/kg), Mbour (2.3 *µ*g/kg), Thiaroye (2.3 *µ*g/kg), Boudody (28.4 *µ*g/kg), Cap Skiring (16.9 *µ*g/kg), Diaobé (5.2 *µ*g/kg), Diogue (13.2 *µ*g/kg), Djiffer (33 *µ*g/Kg), Niddior (6.8 *µ*g/kg) and Sedhiou (14.2 *µ*g/kg) are not B(a)P compliant. The highest concentration of this molecule was observed in Djiffer with a value of 33 *µ*g/kg. However, these values are lower than those obtained by [8] on smoked fish species from south Nigeria, the levels of which were estimated at 204 *µ*g/kg for *Clarias gariepinus* and 288 *µ*g/kg for the species *Ethmalosa fimbriata*. The mean value (8.0 *µ*g/kg) of B(a)P observed in the samples is much higher than that found in [11] whose concentration was below the detection limit (0.01 *µ*g/kg) but lower than that (52.7 *µ*g/kg) obtained in [17] on smoked fish species.

The analysis of samples of smoked fish from various sites also revealed the presence of chrysene at variable concentration levels between 1.3 and 142.6 *µ*g/kg. These were observed in samples from Cayar and Boudody, respectively. The average chrysene content is 35.1 *µ*g/kg. The latter is higher than the mean values of B(a)A and B(a)P. Compared to the results in [18], a content of 69.7 *µ*g/kg of chrysene was obtained in a species of smoked fish called *Thunnus albacares*.

Regarding B(b)F and B(a)A, the levels in the various samples analysed vary, respectively, from 1.7 to 822.3 *µ*g/kg and from 1.2 to 121.6 *µ*g/kg. The average contents of B(b)F and B(a)A, in the samples, were respectively evaluated at 120.1 and 26.4 *µ*g/kg. Much lower levels were observed in [19] on several species of smoked fish, in the Beninese Coast. The average concentrations of B(a)A and B(b)F in these species were estimated, respectively, at 9.99 *µ*g/kg and 5.79 *µ*g/kg.

The total concentration of the four (4) molecules varies between 4.8 and 1082.3 *µ*g/kg, respectively, observed in the samples from Cayar and Diaobé. Higher concentrations of the sum of PAHs, between 1295 and 2020 *µ*g/kg, were obtained in [20]. The average content of ∑4PAHSs in the different samples studied is much higher than that obtained in [11] which was estimated at 12.47 *µ*g/kg. The total PAH content recommended by the European Commission is 12 *µ*g/kg. Thus, only the samples taken in the sites of Cayar (4.8 *µ*g/kg), Dionewar (8.4 *µ*g/kg), and Fass Boye (8.3 *µ*g/kg), with values below the standard, are therefore compliant.

It is clear that also samples from sites, such as Boudody, Dioabé, Diogue, Djiffer, Sedhiou, and Cap Skiring, have the highest PAH contents. The latter has been observed in species such as Kong (*Arius heudelotii*) and Cobo (*Ethmalosa fimbriata*).

### 3.3. Principal Component Analysis

Principal component analysis (PCA) was carried out to assess the effects of smoking, the nature of the fish species, and the place of processing on PAH levels. The first two dimensions (Dim 1 and Dim 2) express 98.90% of the total inertia (Table 4).

The first dimension (Dim 1) contributes to 81.08% and the second (Dim 2) at 17.82%. They thus present the highest eigenvalues, respectively, equal to 3.24 and 0.71.

**Figure 2:**
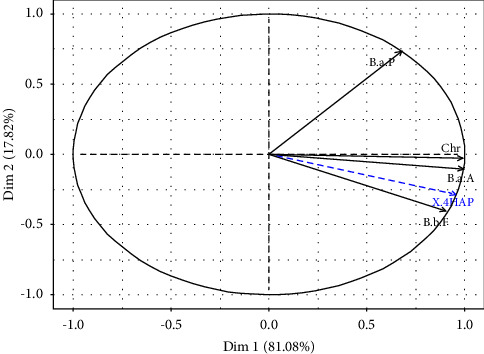
Correlation between PAH contents and the first two dimensions of the PCA.

**Figure 3:**
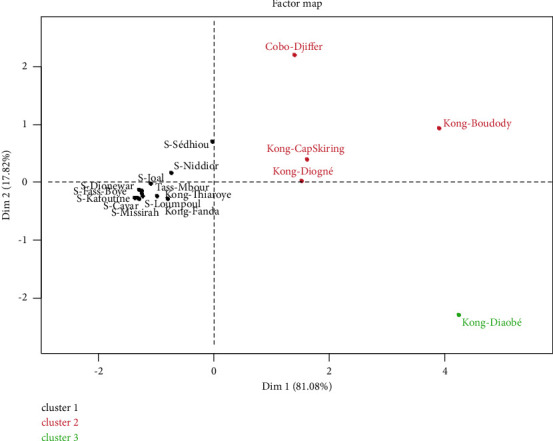
Projection of the different types of smoked fish in the PCA factorial plan.

The variables B(a)A (0.986), B(b)F (0.818), and Chr (0.981) are positively well correlated with the first dimension (Dim 1). However, the second dimension is characterized by the variable B(a)P (0.539), which is positively correlated. Smoked fish have been grouped into three classes. Class 1 is made up of *sardinella* which have very low levels of B(a)P, B(b)F, B(a)A, and Chr. However, high levels of B(a)P and Chr characterize class 2 consisting of smoked fish of the Kong species, from Cap Skiring, Diogue, and Boudody, and of the Cobo species from Djiffer (Figures 2 and 3). The third class, which represents smoked fish of the Kong species from Diaobé, is characterized by B(b)F and ∑4PAH high. In short, based on the standards in terms of PAH content limits for smoked fish, it appears that smoked fish of the *sardinella* species are less carcinogenic for human consumption.

## 4. Discussion

The analysis of the results of this study shows that the B(a)P content and the sum of the ∑4PAHS contents in different zones and according to the species present a significant difference compared to the standard.

This trend could be attributed, on the one hand, to the differences in fat and moisture composition of each species as well as to the nature of the skin cover [21]. Indeed, according to [20], smoked fatty fish are more exposed to the development of PAHs than lean fish. Fat is associated with the pyro synthesis of PAHs. It would also be linked to the traditional methods of smoking carried out in these areas [22, 23]. Indeed, smoking is done exclusively by women, and it is clear that they very often use wood lint or coconut shell as their main fuels. The pyrolysis of cellulose and lignin contained in the wood gives rise to the production of PAHs [24, 25]. Furthermore, the type of wood has a significant influence on the increase of PAH contents in smoked foods. Similarly, the use of cartons during the smoking process could modify the photosensitive properties of PAHs and cause their accumulation in foods [26]. In addition, this is the fact that the PAH content in smoked products varies and depends on environmental factors such as the presence or absence of light or oxygen. In addition, the long smoking times as in the species (*Arius heudelotii*), the temperature used and multiple smoking could lead to the high numbers obtained. Moreover, studies have shown that a smoking temperature above 450°C would allow the pyrolysis of the wood to generate more (PAH) [17, 27].

PAHs would also be present in fish products before smoking. Indeed, apart from spatiotemporal parameters, it has been revealed that the levels of PAHs would also be affected by several ecological factors. It has been demonstrated that vehicles, especially diesel engines, could also contribute significantly to the generation and release of PAHs into the environment [28]. In addition, studies have shown the presence of PAHs in marine sediments which could contaminate the fish before smoking [29]. The combined influence of all these variability parameters associated with possible environmental contamination would certainly constitute the source of the increase in PAH levels in these smoked fish. Thereby, the high levels of PAHs observed in certain areas of this study would probably be linked to ignorance of the existence of the production of PAHs during technological treatment and ignorance of the processes minimizing the production of PAHs. So, the adoption and use of appropriate facilities as studied in [30, 31] are required to considerably reduce the PAH content in smoked fish and to limit the contamination of finished products.

## 5. Conclusion

This study consisted of highlighting the presence of polycyclic aromatic hydrocarbons (PAHs) in traditionally smoked fish. Indeed, smoking, while helping to preserve fish, could also induce certain carcinogenic substances such as PAHs in processed fish. Sampling, carried out in seventeen (17) production areas in Senegal, revealed the presence of benzo(a)pyrene (B(a)P), benzo(a)anthracene (B(a)A), benzo(b)fluoranthene (B(b)F), and chrysene molecules (Chr). The results showed that twelve (12) or 71% and fourteen (14) sites or 82% present contents higher than the European Commission standards, respectively, for B(a)P and the sum of ∑4PAH. The highest PAH concentrations were observed in the Kong species (*Arius heudelotii*). These results, therefore, indicate that it is necessary to improve the smoking process in order to best limit the rate of contamination of the finished products with PAHs and ensure consumer safety. Moreover, it would be very important to carry out an analysis to assess the possible risks to human health associated with the consumption of these smoked fish in Senegal.

## Figures and Tables

**Table 1:** The limit of detection (LOD), limit of quantification (LOQ), linearity, and retention times of the four standard PAHs.

PAHs	Calibration equations	Correlation coefficients (*R*^2^)	Retention times in minutes	Limit of detection (LOD) (*μ*g/kg)	Limit of quantification (LOQ) (*μ*g/kg)
B(b)F	*y* = 28.015*x* + 2.5925	0.9994	24.43	0.05	0.19
B(a)A	*y* = 24.994*x* − 0.1076	0.9997	21.22	0.09	0.24
Chr	*y* = 27.82*x* + 1.5568	0.9995	22.66	0.07	0.20
BaaP	*y* = 24.535*x* + 1.9721	0.9999	26.52	0.08	0.23

**Table 2:** Intraday and interday of precision of the four PAHs.

PAHs	CV (coefficient of variation in %)	
	Intraday	Interday
B(b)F	2.25	3.13
B(a)A	2.17	2.88
Chr	3.33	3.74
BaaP	1.36	2.95

**Table 3:** PAH contents in seventeen (17) samples of smoked fish.

No.	Species	Origins			B(b)F (*µ*g/kg)	B(a)A (*µ*g/kg)	Chr (*µ*g/kg)	B(a)P (*µ*g/kg)	∑4PAH (*µ*g/kg)
1	Kong	Boudody			357.8	107.2	142.6	28.4	636.0
2	Kong	Cap Skiring			236.5	57.2	78.1	16.9	388.7
3	*Sardinella*	Cayar			1.7	1.2	1.3	0.6	4.8
4	Kong	Diaobé			822.3	121.6	133.2	5.2	1082.3
5	Kong	Diogue			249.5	59.5	76.2	13.2	398.4
6	*Sardinella*	Dionewar			3.3	1.3	1.5	2.3	8.4
7	Cobo	Djiffer			116.6	41.7	51.5	33.0	242.8
8	Kong	Fanda			46.4	8.9	23.9	2.2	81.4
9	*Sardinella*	Fass Boye			2.8	2.0	2.7	0.8	8.3
10	*Sardinella*	Joel			15.7	4.3	7.2	3.9	31.1
11	*Sardinella*	Kafoutine			11.4	2.4	6.7	1.4	21.9
12	*Sardinella*	Loumpoul			11.2	1.6	4.9	1.8	19.5
13	Tass	Mbour			10.5	2.4	2.6	2.3	17.8
14	*Sardinella*	Missirah			8.2	3.2	2.6	0.8	14.8
15	*Sardinella*	Niddior			37.1	8.8	16.3	6.8	69.0
16	*Sardinella*	Sedhiou			70.1	20.5	30.4	14.2	135.2
17	Kong	Thiaroye			40.1	5.6	14.3	2.3	62.3
Mean					120.1	26.4	35.1	8.0	161.7

**Table 4:** Correlation between components and variables.

Variables	Main components	
	Dimension 1	Dimension 2
B(b)F	**0.818**	0.163
B(a)A	**0.986**	0.011
Chr	**0.981**	0.001
B(a)P	0.457	**0.539**
Own value	3.24	0.71
Variance (%)	81.08	17.82
Cumulative variance (%)	81.08	98.90

Bold values represent the best correlation coefficients of each variable with respect to the dimensions (1 and 2).

## Data Availability

The data used to support the findings of this study are available from the corresponding author upon request.
